# Light Primes the Escape Response of the Calanoid Copepod, *Calanus finmarchicus*

**DOI:** 10.1371/journal.pone.0039594

**Published:** 2012-06-27

**Authors:** David M. Fields, Steven D. Shema, Howard I. Browman, Thomas Q. Browne, Anne Berit Skiftesvik

**Affiliations:** 1 Bigelow Laboratory for Ocean Sciences, West Boothbay Harbor, Maine, United States of America; 2 Institute of Marine Research, Austevoll Research Station, Storebø, Norway; University of Connecticut, United States of America

## Abstract

The timing and magnitude of an escape reaction is often the determining factor governing a copepod’s success at avoiding predation. Copepods initiate rapid and directed escapes in response to fluid signals created by predators; however little is known about how copepods modulate their behavior in response to additional sensory input. This study investigates the effect of light level on the escape behavior of *Calanus finmarchicus*. A siphon flow was used to generate a consistent fluid signal and the behavioral threshold and magnitude of the escape response was quantified in the dark and in the light. The results show that *C. finmarchicus* initiated their escape reaction further from the siphon and traveled with greater speed in the light than in the dark. However, no difference was found in the escape distance. These results suggest that copepods use information derived from multiple sensory inputs to modulate the sensitivity and strength of the escape in response to an increase risk of predation. Population and IBM models that predict optimal vertical distributions of copepods in response to visual predators need to consider changes in the copepod's behavioral thresholds when predicting predation risk within the water column.

## Introduction

For a predator, an unsuccessful predation event results only in its continued hunger [1]. For the prey, however, an unsuccessful escape response can result in injury or death. In response to such a strong selective pressure, prey have evolved an array of behavioral, sensory and mechanical mechanisms to minimize the risk of predation.

Copepods are heavily preyed upon by visual predators, mainly fish [2]. In response to potential predatory threats, copepods exhibit a rapid and directed escape reaction. Calanoid copepods have evolved very effective escape reactions in response to predators [3], [4]. However, few studies address the stimulus thresholds needed to elicit the escape reaction in copepods and/or how they are modulated by environmental factors such as light.

The biological and physical environment of pelagic copepods requires highly discriminate and yet rapid behavioral responses. Living at low Reynolds numbers, chemical stimuli are transported to the copepod's sensors largely through the slow process of laminar fluid displacement and Fickian diffusion [5]. Similarly, mechanical stimuli are attenuated quickly by viscous dampening causing fluid velocity to decrease with distance cubed [6]. Because these signals attenuate rapidly with distance, copepods often do not detect other individuals until they are within a few body lengths and have, therefore, evolved mechanisms to maximize their ability to detect predators [7], [8], [9], minimize their behavioral latencies [10], and achieve extraordinarily rapid escape velocities [3], [4], [11], [12].

Mechanoreception is a primary mechanism for the remote detection and discrimination of predators [6], [9], [13], [14]. Numerous mechanoreceptive setae populate the first antennae of copepods providing a wide range in sensitivity to fluid mechanical signals [14], [15], [16], [17]). Escape behaviors appear to be initiated in response to relatively few neural signals [17] initiated by sensor displacements of as little as 10 nm [13], [14]. Transmission speed from the sensor to the motor neurons is augmented by myelin-like structures along the neurons, permitting behavioral responses within ms of signal generation [10]. Despite the extreme sensitivity of individual mechanosensory setae, copepods rarely respond behaviorally to these small fluid disturbances. Thresholds for initiating behavioral responses are often orders of magnitude higher than neurophysiological thresholds [6], suggesting that copepods can modulate their responses based on perceived risk.

In addition to mechanical signals, copepods also detect and respond to light [18]. Much of the work investigating the response of copepods to light has involved flashing stimuli as a mechanism to directly stimulate copepod behavior [19], [20]. This is in contrast to most ecological situations, where light levels remain relatively constant. Under these conditions, light intensity or gradients in light levels are unlikely to act as the proximate cue driving the initiation of the rapid escape reaction but may modulate the behavioral sensitivity of copepods to other sensory cues, including fish kairomones [21] and mechanical signals. Little is known about the interactive effects of visual and mechanosensory stimulation on copepods. However, since visual predators attack copepods more often and at greater distances in the light rather than in the dark [22], [23] it is reasonable to hypothesize that copepods alter their behavioral sensitivity to mechanosensory stimulation when light levels favor the success of visual predators.

*Calanus finmarchicus* are prey to visual predators including fish [24] and krill [23]. In this study a siphon flow was used to investigate the behavioral sensitivity of *Calanus finmarchicus* CV and adult stages to fluid mechanical signals in the light and dark. We hypothesized that, in response to the higher predation risk from visual predators in the light, *C. finmarchicus* will initiate an escape reaction at a lower threshold (further from the source) in the light than in the dark. In addition to the lower behavioral threshold, the magnitude of the escape response was hypothesized to be greater when the perceived predation threat was higher.

## Methods

### Test animals

Copepods (*Calanus finmarchicus*) were cultured in large 5000 L flow-through silos at the Institute of Marine Research's Austevoll Research Station, Norway. Animals were maintained at 12.5°C on a mixed diet of *Rhodomonas baltica*, and *Isochrisis sp*. at a food level of 2×10^4^ cells mL^−1^. Individual adult stage copepods were collected in a large beaker and held for ∼2 hours in 20 L buckets at 12.5°C in the dark prior to testing. Copepods (100–150 per treatment) were placed within the filming vessel (tank size 25 cm ×25 cm ×60 cm; 37.5 L) and allowed to acclimate to the test condition for 10 minutes. Animals were filmed for 30 minutes. To test if light levels modulate copepod escape characteristics, a constant fluid mechanical signal was maintained and tested the response of copepods in the dark and at light levels found at 20 m during an average Bergen, Norway summer (see below).

### Siphon Tank Configuration

**Figure 1:**
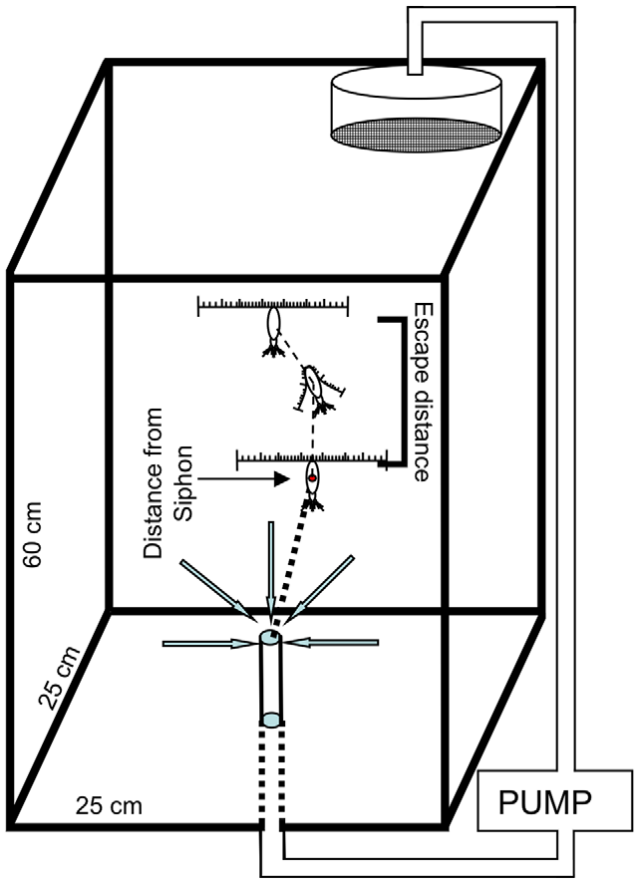
Filming apparatus used to examine the escape reaction of *Calanus finmarchicus* in response to a suction flow. The tank volume was 37 L (25×25×60 cm). The total volume viewed using this optical setup was ∼2.5 L surrounding the siphon (10×10×25 cm). Red dot in the center of the animal indicates the data point taken from the video frame just prior to the initiation of the escape reaction. The distance of copepod from the siphon at the initiation of the escape response represents the detection threshold. Once the animal initiated the escape reaction (retracts its antennae), the total distance traveled and the speed of the escape were measured.

A siphon flow was used to create a stable fluid mechanical disturbance. The resulting flow fields are well-characterized and have been used to analyze copepod escape behavior [25], [26], [27]. The flow was created by a gravity-forced drain through a 16-gauge, stainless steel, flat-tip hypodermic needle mounted 70 mm above the bottom of the tank. The flow rate exiting the tank was 2.0 mL s^−1^. A constant head pressure was maintained by simultaneously returning the drained water to the top of the tank. To diminish the disturbance to the calibrated flow field created by the siphon, incoming water was pumped back into the tank through a 105 mm diameter vessel with a 35 µm mesh screen located just below the water's surface (Fig. 1). The experiment was conducted in a climate controlled room at 12.5 (**±**0.5) °C. Each experimental condition was run in triplicate with 100–150 animals per replicate (2.6–4.0 animals L^−1^). Each replicate was filmed for 30 minutes. Animals were not used more than once.

### Light signal

Copepod escape responses were observed under two light settings (changed using quartz substrate neutral density filters applied to the collimated output of a 1000 W Xenon arc lamp). The light beam was positioned above the tank to produce a homogeneous light field at the top of the filming vessel. Light levels used in these experiments simulated dusk (1.07×10^−4^ W cm^−2^) and darkness at a depth of ∼20 meters [28], corresponding to the upper range of distribution of *Calanus finmarchicus* in the fiords outside of Bergen Norway during mid Spring.

### Video observations and analyses

Silhouette imaging was used to observe copepod behaviour in 3D. The optical setup provides fine-scale behavioral observations with an image quality that is unaffected by ambient light levels (described in [29]). In short, the system consists of two orthogonally-oriented cameras with a far red light emitting diode (LED) placed at the focal point of a biconvex collimating lens, the output beam (15 cm diameter) of which passes through an aquarium (25×25×60 cm) placed at the intersection. Video images are collected at 25 hz. The total volume viewed using this optical setup is ∼2.5 L surrounding the siphon (10×10×25 cm). The velocity (V) of the water entrained by the siphon decreases exponentially with distance (r) from the siphon as:

V = Q (4π r^2^)^−1^where Q is the volume exiting the siphon [26]. At edges of the viewing area (5 cm from the siphon) the flow created by the siphon is 60 um s^−1^. This is well below the escape threshold for most species [9] and near the neurophysiological threshold for detection [14].

Animal position, speed and distance travelled were measured using custom designed software packages (Measure, by JASCO Research; described in [30]).

### Behavioral Analyses

The threshold and magnitude of escape response of CV and adult stage *Calanus finmarchicus* was quantified using three characteristics of their escape reaction. To determine the behavioral threshold of the escape response, we measured the distance from the flow source (suction flow: see below) at which the copepod initiated an escape reaction. Once initiated, the magnitude of the escape reaction was assessed by the measuring the average speed of the entire escape reaction and the total distance traveled during the escape.

The appendages (and their motion) involved in an escape reaction have been described for *Cyclops sp*. [3], [10], [31] and *Oithona sp* [32] and can easily be differentiated from a simple flick response or an attack response [6] based on the appendages used. The escape reaction can involve a single jump during which the antennae are drawn to the sides of the body followed by the motion of the swimming legs or a series of jumps in which there is one beat of the first antenna followed by multiple cycles of motion in the swimming legs [3], [32]. Both single and multiple jumps (from a single escape) were quantified in this analysis. Since the threshold for the escape reaction decreases with multiple sequential escapes [32], in cases where the flow re-entrained the same animal after an escape, only the first escape reaction was used for further analysis. Escape reactions that occurred below the mouth of the siphon, or whose location was obstructed by another animal in one of the views, were not used in this analysis. The escape distance was calculated as the cumulative distance traveled over the entire escape sequence. The distance was calculated at 40 ms intervals to capture the total length of a tortuous path. The speed of the escape reaction was calculated as the total distance traveled during the escape response divided by the duration of the entire escape reaction.

### Statistical Analysis

The distances from the siphon at which *Calanus finmarchicus* initiated the escape reaction (threshold) and the total distance traveled during the escape reaction were not normally distributed. Therefore, differences in the threshold distance and travel distance were analyzed using the non-parametric Mann-Whitney ranked sum test. The escape speeds, which were normally distributed, were analyzed using a 2 tailed t-test.

## Results

The escape characteristics of a total of 116 copepods were examined at the two light levels. Each replicate tank produced between 15–26 escape reactions in both the light and dark treatments. No significant difference was found between replicates within the same treatment and they were, therefore, pooled for further analyses. All animals entrained by the siphon initiated an escape reaction. Two copepods initiated an escape reaction but were captured by the siphon. The escape characteristics of these animals were not analyzed as part of this study.

### Escape Sensitivity

**Figure 2:**
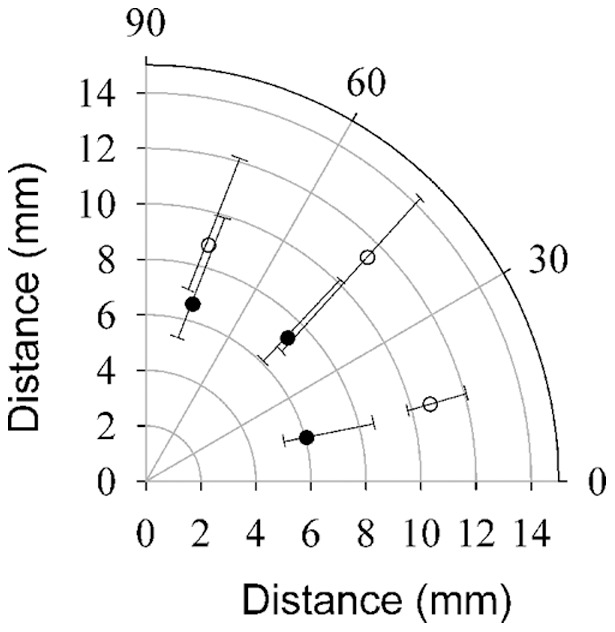
Median distance from the siphon at which *Calanus finmarchicus* initiated an escape reaction in the dark (solid circles) and in the light (open circles). Lower and upper whiskers represent the 25% and 75% distribution. See table 1 and 2 for data and statistics for light and dark treatments, respectively.

**Figure 3:**
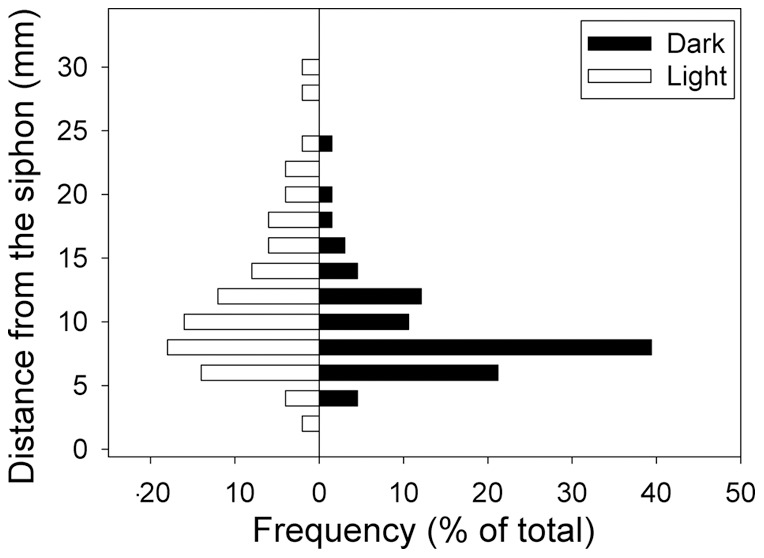
Escape distributions of *Calanus finmarchicus* from a siphon flow. Frequency has been normalized to total number of escapes for each treatment; n = 66 for the dark and n = 50 in the light. Distribution were tested on raw data using a Mann-Whitney ranked sum test and found to be significantly different. U Statistic  = 1230.5 (P = 0.019).

The behavioral threshold for *C. finmarchicus* to the fluid mechanical signal was quantified as the distance from the siphon at which the animals initiated their escape reaction (Fig. 1). The distribution of escape reactions surrounding the siphon were laterally symmetrical in both the light and the dark treatments and were, therefore, transposed into a single quadrant for further analysis (Fig. 2). Within each treatment, the escape distances did not differ with respect to the angle of entrainment relative to the siphon (Tables 1 and 2). However, comparison of the escape reactions in light and dark treatments showed significant differences (Table 3; Fig. 3). *C. finmarchicus* initiated their escape reactions significantly further from the siphon flow in the light. The median value for the escape distance was 6.9 mm (3.1 body lengths; BL) from the siphon mouth in the dark and 9.4 mm (4.2 BL) in the light; an increase of 36%.

**Table 1:** Distance from the siphon at which *Calanus finmarchicus* initiated an escape reaction in the dark as a function of angle.

Angle	N	Median	25%	75%
15	6	6.1	5.4	7.6
45	29	7.3	6.4	10.2
75	31	6.6	5.9	10.0

Data was tested using Kruskal-Wallis One Way Analysis of Variance on Ranks. H = 2.406 with 2 degrees of freedom. (P = 0.300).

**Table 2:** Distance from the siphon at which *Calanus finmarchicus* initiated an escape reaction in the light as a function of angle.

Angle	N	Median	25%	75%
15	4	10.7	8.2	12.1
45	21	11.4	6.8	15.1
75	25	8.8	6.0	12.4

Data was tested using Kruskal-Wallis One Way Analysis of Variance on Ranks. H = 1.100 with 2 degrees of freedom. (P = 0.577).

**Table 3:** Distance at which *Calanus finmarchicus* initiated an escape reaction from the siphon in the dark and light.

Treatment	N	Median	25%	75%
Dark	66	6.9	6.0	9.9
Light	50	9.4	6.4	14.1

Data tested with a Mann-Whitney ranked sum test. U Statistic  = 1230.5 (p = 0.019).

### Strength of the escape reaction

**Figure 4:**
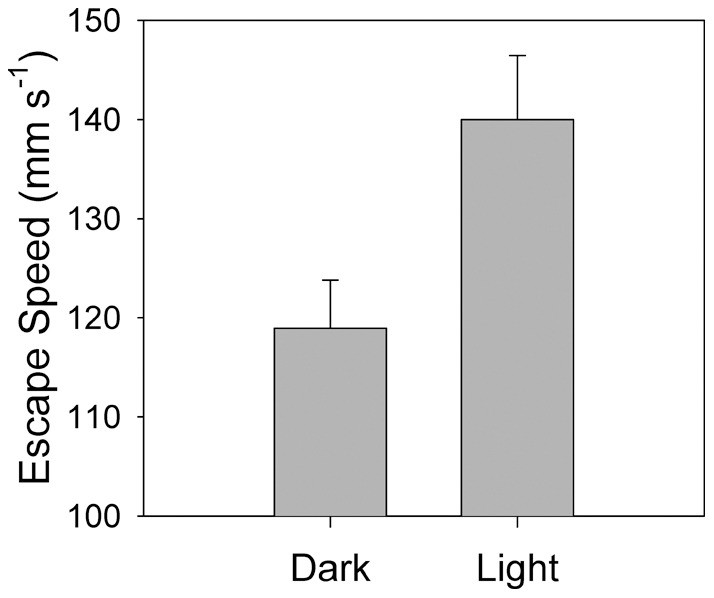
Average escape speed of *Calanus finmarchicus* from a siphon flow (± standard error of the mean). Mean values are significantly different (p = 0.01; n = 66 for the dark and n = 50 in the light).

Once the escape reaction was initiated, the strength of the response was quantified by the speed of the escape and total distance traveled. Escape speed of *C. finmarchicus* was significantly faster in the light than in the dark (Table 4; Fig. 4). In the dark, the average escape speed was 119 mm s^−1^ (**±**53.6 BL s^−1^). In the light, the average escape speed was 140 mm s^−1^ (**±**63.6 BL s^−1^), an increase of 18% compared to the escape speeds in the dark. The higher escape speeds, however, did not result in a greater distance traveled during the escape (Table 5). Median value for the escape distance in the dark was 16.7 mm (7.6 body lengths; BL) and 14.7 mm (6.7 BL) in the light. Distance from the siphon at which the copepod initiated the escape reaction explained only a small fraction of the variation in either the speed of the escape reaction (r^2^<0.01) or the total distance traveled (r^2^<0.06) during the escape.

**Table 4:** Escape speed of *Calanus finmarchicus* from the siphon in the dark and light.

Treatment	N	Mean (± STD)	SEM
Dark	66	119.0 (39.2)	4.8
Light	50	140.0 (45.6)	6.5

Data tested with standard t-test. t = 2.67 (p = 0.009)

**Table 5:** Escape distance of *Calanus finmarchicus* from the siphon in the dark and light.

Treatment	N	Median	25%	75%
Dark	66	16.7	11.4	26.4
Light	50	14.7	11.4	29.8

Data tested with a Mann-Whitney ranked sum test. U Statistic  = 1644.5 (p = 0.98).

## Discussion

The distance from the predator at which an organism initiates an escape response and the strength of the escape (speed and distance traveled) can be the determining factors governing an organism's ability to avoid predation [9], [33]. While it is known that copepods modulate their activity with changes in perceived predation risk and food availability [34], [35], [36], [37], [38], this is the first study to examine the effects of ambient light levels on the sensitivity and magnitude of their escape reaction.

Virtually all copepods exhibit an escape reaction in response to a perceived predation threat. Consistent with earlier studies [9], [26], our data shows that copepods remotely detect the fluid mechanical disturbance generated by the siphon and initiate an escape reaction in response to it. The distance from the siphon at which *Calanus finmarchicus* initiated the escape reaction showed no significant difference with respect to the angle from the siphon (Table 1–2; Fig. 2). These results are inconsistent with the data reported for *Acartia tonsa* [39] which showed the greatest sensitivity when approached laterally by the suction flow. Differences in antennule architecture may provide part of the answer to this inconsistency. The setal array on the antennules of *Calanus finmarchicus* are organized linearly along the antennule with nearly all of the mechanosensory setae pointing anterior to the animal. *Acartia tonsa*, in contrast, has setae surrounding the axis of the antennule, potentially providing much greater three dimensional spatial resolution of surrounding fluid motion.

The behavioral sensitivity (Fig. 3, Table 3) and the magnitude (Fig. 4, Table 4) of the escape reactions undertaken by *Calanus finmarchicus* were higher in the light relative to the dark treatment. These results are consistent with previous studies that demonstrated the synergistic effects of different sensory cues on the behavioral response of marine crustaceans [21], [40]. For visual predators, the greater the intensity of the light, the further the predators can see their prey and initiate an attack [41], [42], [43]. Since light intensity decays exponentially with depth, predation risk has been hypothesized and modeled to show a similar decline (e.g. [44]). Experiments on feeding in fish demonstrate that the light level and optical properties of the water determine the likelihood that prey is detected and consumed [11], [45]. The vision-based predation model of Eggers [46], and later models (e.g. [47], [48], [49], concluded that correctly incorporating the optical environment is essential to predicting the outcome of visual predation. However, these results are consistently based on the assumption that the escape response of prey is constant, an assumption that is falsified by the results of this study.

**Figure 5:**
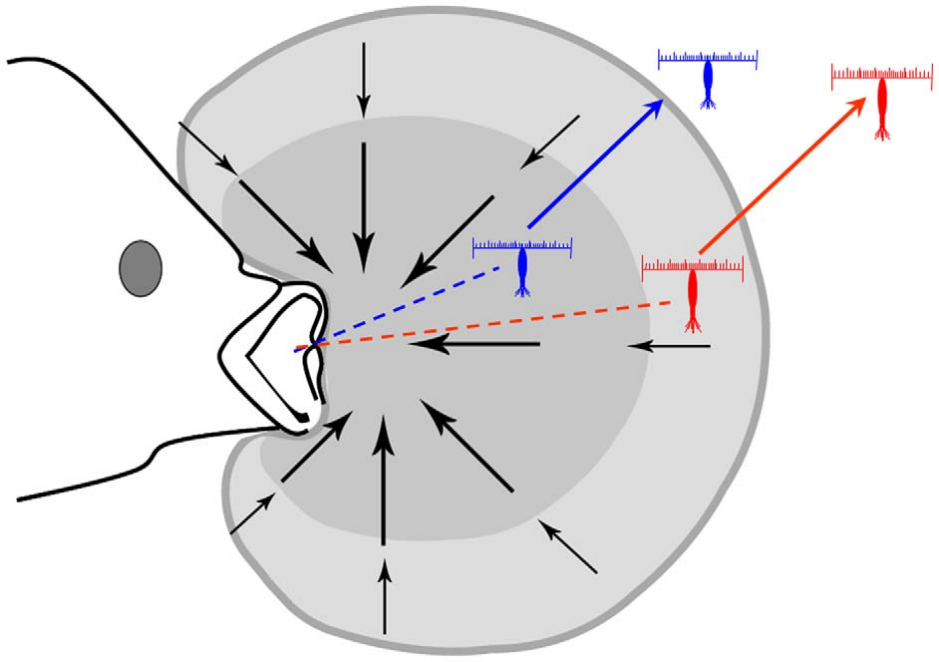
Hypothetical predatory interaction between a planktivorous fish and their copepod prey. Shaded region represents the maximum volume of water entrained during a single strike. The dark shaded area represents the region where the fluid mechanical threshold evokes an escape reaction by the copepod in the dark. The light shaded region represents the location where the escape reaction is initiated in the light. In both cases, the distance traveled during the escape is sufficient to remove the copepod from the capture volume.

When feeding, planktivorous fish entrain a discrete volume of fluid during each strike (Fig. 5). A subset of the entrained volume enters the buccal cavity. Although there is only limited data available quantifying the volume engulfed by different sizes or species of fish, Day et al, [50] estimates that a 15 cm bluegill sunfish ingests a volume of fluid (capture volume) ranging from 1.8 to 6.5 mL during a single strike. Assuming a sphere surrounding the mouth of the fish, the lateral extent of this volume is ∼7.5 to 11.6 mm from the mouth of the fish. Copepods that are further away (outside of the capture volume) are not at risk of being consumed during the predatory attack, although they may be entrained by the fluid. These copepods do not need to initiate an escape reaction. In contrast, copepods within the capture volume need to initiate an escape reaction to avoid being consumed. As the fish begins to engulf the fluid, the outer edge of the volume moves inward and the speed of fluid within the capture volume gets progressively faster. Thus, by waiting, the copepod decreases the distance that they need to travel during the escape but they must increase the velocity needed to escape entrainment. On average, copepods initiate an escape reaction when they were 6.9 mm and 9.4 mm from the siphon in the light and dark respectively, falling just within the hypothetical capture volume of the bluegill sunfish. The average escape distance of *C. finmarchicus* (20.9 mm) would transport the copepod well outside this capture volume providing the copepod a temporary reprieve from the threat of predation. The risk of a second attack would depend in part on the visual acuity of the predator, light level and the optical quality of the water ([51] and refs therein).

Fluid mechanical disturbances become neurological signals through the motion of one or several of the numerous mechanoreceptive setae that adorn the antennules of copepods [13], [15], [17]. The fluid speed needed for the escape reaction (behavioral threshold) is orders of magnitude higher than the fluid speed needed to generate a neurophysiological signal. Neurophysiological data suggest that individual mechanoreceptors of copepods are sensitive to nanometer displacements and can detect fluid velocities as low as 20 µm s^−1^ [13]. If eliciting the earliest escape reaction possible was the only factor determining the threshold at which prey initiate an escape reaction, the escape would occur when fluid speeds exceed 20 µm s^−1^. At flow rates used in this study (∼2 mL s^−1^), escape reactions would occur at 125 mm (56BL) from the siphon. Assuming that the neurophysiological thresholds are similar to those reported above, *C. finmarchicus* do not escape at their neurophysiological threshold but rather initiate escapes only in response to much higher signal strength. The intermediate value of the escape threshold suggests two opposing forces which determine the upper and lower limits of the magnitude of the stimulus needed to cause an escape reaction [9]. The upper limit is probably defined by the risk of a delayed escape reaction. The strike efficiency of a predator commonly increases with decreased distance from its prey. Therefore, as the predator approaches the copepod, the longer they wait before initiating an escape reaction the higher the probability of being captured. The finality of an unsuccessful escape clearly has strong evolutionary repercussions on the individual and is expected to apply strong selection pressure in shaping the timing and location of the escape reaction.

Less obvious, and more difficult to assess, are the conditions that give rise to an inhibition of the escape reaction despite being within the neurophysiological detection limit of the copepod [36], [52]. There are several possible explanations to account for this discrepancy. The first is that the escape reaction incurs an energetic cost on the copepod, consuming up to 400 times the normal energetic expenditure [3], [53], [54]. With such high energetic costs, it is important that the first escape effectively remove the copepod from the visual field (or at least the strike range) of the predator. Repetitive escapes draw from metabolic reserves causing each sequential escape to be slower and to transport the copepod a shorter distance [32]. Kils [55] showed that exhausted copepods are captured more easily by juvenile herring. Secondly, unnecessary escape reactions increase predation risk by attracting the attention of visual [9], [56], [57] and mechanoreceptive predators [58], [59]. An additional consequence of the rapid escape response is the increased encounter rate with predators that results from the higher swimming speeds [60]. In this context, the escape threshold of the copepod should be a function of the risk of predation due to a delayed escape reaction and the energetic cost and increased predation risk associated with an unnecessary escape [9], [52].

It has long been hypothesized that copepods attempt to decrease predation risk from visual predators by vertically migrating out of the photic zone during the day [61], [62], [63]. Leaving the warm, food-rich photic zone is not without energetic cost, however. As copepods move to colder, food-depleted waters they experience decreased ingestion rates, lower growth rates and ultimately produce fewer eggs (e.g. [64], [65]). Entering the surface water earlier in the day or delaying when they descend could, therefore, provide greater fitness if the risk of predation is diminished [52]. Indirect effects of predation can have enormous impact on the reproductive outcomes of invertebrate prey [66]. An adaptive escape threshold that varies with changing predation risk provides a potential mechanism for copepods to extend their stay in the surface water. Modeling optimal behavior has become a fashionable tool for interpreting distribution patterns of zooplankton. Although most models incorporate vertical migration of copepods in response to light in their calculations, the escape characteristics to visual predators are always held constant. By modulating the escape reactions toward higher sensitivity and greater escape magnitude, copepods may be able to stay further up in the water column than previously assumed. Empirical observations such as those reported here are essential to accurately parameterize individual based ecological models [49], [67] and are required to arrive at an intuitive mechanistic understanding of trophic interactions.
